# Challenges of providing spiritual care to patients in Iranian teaching hospitals: a qualitative study

**DOI:** 10.1186/s40359-026-04028-0

**Published:** 2026-01-26

**Authors:** Adel Eftekhari, Somayeh Bagheri, Najmeh Baghian

**Affiliations:** 1 Department of Nursing, Meybod School of Medical Sciences, Shahid Sadoughi University of Medical Sciences, Yazd, Iran; 2 Clinical Research Development Center, Shahid Rahnemoon Hospital, Shahid Sadoughi University of Medical Sciences, Yazd, Iran; 3 Islamic Spiritual Health Research Center, Shahid Sadoughi University of Medical Sciences, Yazd, Iran

**Keywords:** Clinical care, Hospital, Patient, Qualitative study, Spiritual care, Spiritual care

## Abstract

**Background:**

Recognizing the spiritual dimension of human existence is crucial to overall physical, mental, and social well-being. Spiritual care, as an essential component of holistic healthcare, has garnered increasing attention from researchers and health professionals in recent years. Despite its significance—particularly in hospital settings aimed at enhancing patients’ quality of life—numerous challenges impede effective implementation. This qualitative study aimed to identify the challenges in providing spiritual care to patients in Iranian teaching hospitals.

**Methods:**

This qualitative study used a conventional content-analysis approach and was conducted in 2024. The study population comprised experts in spiritual care and other stakeholders. Data were collected through semi-structured interviews with 24 participants selected via purposive sampling. MAXQDA (version 22) software was used to manage and analyze the data. Each interview transcript was coded using open, axial, and selective coding.

**Results:**

The study identified key challenges in providing spiritual care within medical centers, categorized as: lack of belief (lack of knowledge and awareness; challenges of contextualization); inefficiency in resource management (infrastructure challenges; lack of allocation of financial resources; human resource challenges); process challenges (weaknesses in laws and regulations; lack of enforcement guarantee; lack of management stability; lack of a documented M&E system); limitations of program organization (poor intra-sectoral coordination; poor external coordination); and educational challenges (lack of value in the educational system; lack of effective education).

**Conclusions:**

Addressing these identified challenges requires a comprehensive and coordinated approach that considers cultural, economic, legal, and educational dimensions. Developing and promoting a national model for spiritual care, along with conducting regular evaluations, is essential to ensure effective implementation.

**Supplementary Information:**

The online version contains supplementary material available at 10.1186/s40359-026-04028-0.

## Introduction

Health is a comprehensive and holistic concept that encompasses all dimensions of human existence, including physical, psychological, social, and spiritual aspects. Evidence has demonstrated that the spiritual dimension significantly influences other facets of well-being and is considered fundamental to the health of every individual [1]. The definition of Spiritual Care extends beyond mere religious adherence, encompassing the cultivation of a sense of awe and reverence for existence [2]. Spiritual care involves two interrelated dimensions aimed at promoting the patient’s overall Spiritual Health: religious health (connection to a Divine Power or ultimate reality) and existential health (relationship with others, self, and the environment) [3, 4]. Spiritual care in a clinical context, however, goes beyond addressing an individual’s personal spirituality; it involves the practical application of spiritual principles and practices by healthcare professionals to support patients’ spiritual needs during illness or distress. It encompasses providing comfort, hope, meaning, and connection to patients and their families, respecting their beliefs, and facilitating spiritual growth and well-being [5–8].

The recognition of spiritual care as an essential element in healthcare has grown and is increasingly acknowledged as vital for promoting holistic health [3, 6]. Spiritual care is now considered an integral part of patient-centered care, aiming to address the whole person—body, mind, and spirit. Meeting the spiritual needs of patients and their families is regarded as a critical component of clinical care [9]. When a person is diagnosed with a disease, they often face mental and psychological challenges, experience diminished roles and activities, and undergo changes in social relationships, leading to a heightened search for meaning and purpose in life. Consequently, their need for spiritual care becomes more pronounced [5, 10]. In situations of illness, particularly with chronic or terminal diseases, spiritually focused care has been emphasized by healthcare providers [6–8]. Various studies indicate that spiritual care and spiritual care positively impact quality of life and social well-being and help alleviate symptoms of chronic physical and mental illnesses [9–13]. For instance, Sankhe et al. (2020) found that patients who received spiritual care reported lower levels of anxiety and depression compared with those who did not [9].

Despite its recognized importance, the practical implementation of spiritual care remains neglected in many healthcare settings, leading to spiritual distress, isolation, and reduced hope [5, 14]. Its integration necessitates enhanced provider training, the formulation of clear protocols for spiritual assessment, and the development of effective spiritual support programs, alongside fostering an organizational culture that values spiritual well-being. While previous research has emphasized the positive effects of Spiritual Care [7, 8, 15], a significant gap persists concerning the specific challenges of implementing spiritual care within hospital environments and identifying effective strategies to overcome these barriers. Moreover, there is limited qualitative research exploring the lived experiences of healthcare providers (HCPs) in delivering spiritual care and the barriers they encounter. Exploring these experiences is crucial because HCPs are at the forefront of providing spiritual care, and their perspectives can offer valuable insights into practical challenges and strategies for integration into routine practice. This study aims to bridge that gap by using a qualitative design to understand the challenges faced by HCPs in implementing spiritual care [16–19].

Spiritual care has a substantial impact on coordinating and balancing multiple health dimensions. It also affects patients’ quality of life and longevity. Therefore, it is essential to address the challenges of integrating spiritual care into the healthcare system, especially in hospitals. Consequently, this study aims to explore the implementation challenges of spiritual care in the teaching hospitals of Shahid Sadoughi University of Medical Sciences in Yazd. This context is particularly relevant given its unique cultural and religious environment, where traditional values and beliefs profoundly influence the delivery and acceptance of spiritual care.

The theoretical framework of this study posits that Spiritual Care aligns with psychological theories such as coping, resilience, and disease adaptation, functioning as a cognitive-emotional regulatory mechanism. This meaning-making process mitigates existential distress and enhances self-efficacy, directly contributing to the improvement of Spiritual Health, and is consistent with models like Terror Management Theory and positive psychology. This established link reinforces the theoretical scope of the study by situating it within the broader context of mental and holistic health promotion. These mechanisms correspond with models such as Terror Management Theory and Positive Psychology, whereby spiritual connection enhances perceived control and psychological stability. Thus, integrating spiritual care within hospital settings not only addresses cultural and religious dimensions but also reflects evidence-based psychological constructs underpinning holistic health. Strengthening this link advances the theoretical scope of the present study, situating the findings within broader frameworks of mental-health promotion and resilience-oriented interventions for patients.

Shahid Sadoughi University of Medical Sciences is a major academic medical center in Yazd, Iran, comprising several teaching hospitals that serve a diverse patient population. Understanding the challenges within this specific context can provide valuable insights applicable to similar settings in Iran and other countries with comparable cultural and religious backgrounds.

## Methods

This study adopted a qualitative approach to explore and describe the experiences of healthcare personnel and patients regarding spiritual care. Qualitative research is particularly well suited to investigating complex phenomena from the perspective of those who directly experience them, providing a deeper understanding of the nuances and contextual factors influencing spiritual care within this specific research setting.

The research was conducted in teaching hospitals affiliated with Shahid Sadoughi University of Medical Sciences in Yazd, Iran, including Shahid Rahnomoon, Afshar, and Shahid Sadoughi Hospitals. These teaching hospitals were chosen because they serve as training grounds for future healthcare professionals and are considered key locations for integrating spiritual care into medical education and practice.

### Participants and sampling

Participants were selected through purposive sampling and comprised 24 knowledgeable, informed, and experienced experts in spiritual care and stakeholders in spiritual care delivery. The participant group included doctors, nurses, clergy, spiritual-health professionals in leadership roles, psychologists, healthcare managers, and patients. The operationalization of ‘significant knowledge and experience’ follows: for professionals, a minimum of 5–10 years of relevant specialized experience; for patients, a history of prolonged hospitalization (≥ 10 days) with spiritual needs documented by healthcare staff.

### Data collection

Data were collected through semi-structured interviews. An interview guide was specifically developed, beginning with broad, open-ended questions such as, “What do you think are the most important current challenges in providing spiritual care in the teaching hospitals of Shahid Sadoughi University of Medical Sciences in Yazd?” and “What do you think are the obstacles to the acceptance and implementation of spiritual care by medical staff?” Follow-up and probing questions were used to delve deeper into specific issues and to elicit richer accounts of participants’ experiences.

The average interview duration was 60 min (range: 50–70 min), and interviews were conducted at participants’ workplaces. Data saturation was reached after 20 interviews, with 4 additional interviews conducted to confirm that no significant new themes emerged. Interviews continued until the saturation point was achieved in July 2024.

### Data analysis

Data analysis followed a conventional content-analysis method based on the work of Elo and Kyngäs [20]. After transcription, analysis was conducted concurrently with data collection. A three-stage coding process, drawing on principles of Grounded Theory methodology, was employed:

1. Open coding: Applied line-by-line to extract semantic units and assign descriptive codes.
2. Axial coding: Categories were combined, and relationships between them were determined through the researchers’ interpretive lens.
3. Selective coding: Categories and their relationships were interpreted to articulate and describe emerging themes.

Data management and analysis were facilitated using MAXQDA software (version 22).

### Reflexivity and quality assurance approach

To ensure the trustworthiness of the findings, Lincoln and Guba’s four criteria (credibility, transferability, dependability, and confirmability) were rigorously applied to support findings with documented evidence:

- *Credibility*: Addressed through member checking. A summary of initial results and interpretations was presented to five participants (two patients, two care providers, and one religious leader) to obtain their feedback, which was incorporated into the final analysis.
- *Transferability*: Enhanced by providing rich, detailed descriptions of the research context, participant characteristics, and data-collection processes.
- *Dependability*: Ensured through collaborative data collection and analysis, and by maintaining a comprehensive audit trail documenting all steps and decisions.
- *Confirmability and bias mitigation (dual coding)* : To assess the reliability of the qualitative analysis and mitigate researcher bias, 20% of interviews were independently coded by two coders. The Cohen’s Kappa value was 0.78 (substantial agreement) for initial open coding and 0.85 (almost perfect agreement) for axial coding. Furthermore, two external qualitative-research experts served as secondary coders, and an external data audit was conducted to further enhance confirmability.

## Results

The study included 24 knowledgeable and experienced experts in health and spiritual care, as well as stakeholders from medical centers, including doctors, nurses, and patients (Table 1).

**Table 1:** Information about the interviewees (stakeholders) participating in the study

Field of activity	Academic fields	Number of interviews	Age	Gender	Years of experience
Responsible for spiritual care Ministry of Health	PhD in Quran and Islamic Science Resources	1	54	Male	23
Spiritual care Research Center, Shahid Sadoughi University of Medical Sciences, Yazd	PhD in Epidemiology/PhD in Guidance and Counseling/PhD student at the University of Tehran in Jurisprudence and Fundamentals of Islamic Law	3	60	Male	29
			43	Female	13
			38	Male	10
Office of the University Leadership Institute	PhD of Education, Revolution Orientation, Level 3, Area/Master of Imamiyya Theology and Final Semester PhD Student at the University of Quran and Hadith, Qom	2	45	Male	15
			40	Male	8
Assistant Student Cultural Officer, Shahid Sadoughi University of Medical Sciences, Yazd	PhD of Quran and Hadith, Master of Nahjul Balagha, Level 3	1	48	Male	22
Hospital Director	Medicinæ Doctor, Executive Management	3	55	Male	22
			46	Male	16
			58	Male	25
Health Education and Promotion	PhD in Health Policy/Bachelor of Nursing	2	45	Female	19
			33	Female	11
Nurse	Bachelor of Nursing/Master of Healthcare Services Management	2	44	Female	22
			40	Male	15
physician	Surgery/Oncology Specialist	2	49	Male	14
			46	Female	13
Spiritual Care Expert	Seminary Education	4	46	Male	16
			42	Female	8
			40	Female	10
			36	Female	8
Islamic Psychology	PhD in Psychology and Education of Exceptional Children, Researcher in Islamic Psychology	1	48	Male	20
Patient	Diploma/Bachelor	3	35	Female	10 day length of stay
			28	Female	12 day length of stay
			44	Male	10 day length of stay

**Fig. 1:**
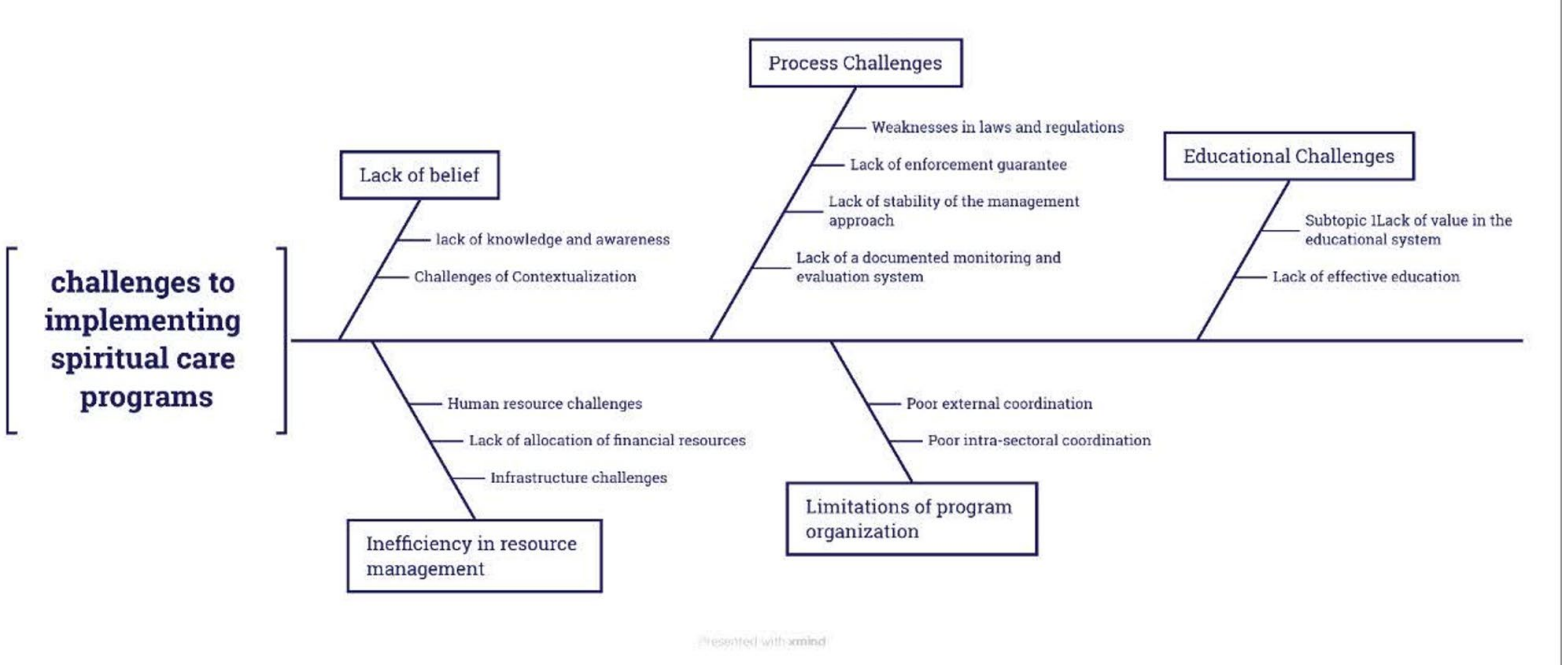
Challenges to implementing spiritual care programs from the perspective of stakeholders

Content analysis of the interviews identified five main categories and thirteen subcategories of challenges. Stakeholders reported key obstacles to implementing spiritual care, categorized as: Lack of belief; Inefficiency in resource management; Process challenges; Limitations in program organization; and Educational challenges (Table 2; Fig. 1).

**Table 2:** Categories and subcategories extracted as challenges to implementing spiritual care programs from the perspective of stakeholders

Theme	Sub-theme	Codes (concise)
Lack of belief	Lack of knowledge & awareness	Misunderstanding of spiritual health; Staff unaware of spiritual care unit role
	Challenges of contextualization	Failure to create cultural context; One-dimensional (biomedical) view of health; Ideological rigidity about spiritual care
Inefficiency in resource management	Infrastructure challenges	Insufficient private/appropriate spaces; Bureaucratic delays for equipment/supplies
	Lack of allocation of financial resources	No dedicated budget for spiritual care; Irregular or insufficient payment to staff
	Human resource challenges	Shortage of qualified clergy/experts; Recruitment and retention difficulties
Process challenges	Weaknesses in laws & regulations	No formal MOH organizational placement; Undefined referral protocols; No dedicated MOH unit
	Lack of enforcement guarantee	Insufficient managerial/administrative support; Irregular policy meetings and follow-up
	Lack of management stability	Program disruption after leadership change; Lack of sustainable organizational culture
	Lack of documented M&E system	No systematic monitoring/evaluation; Lack of documented evidence of program effectiveness
Limitations of program organization	Poor intra-sectoral coordination	Limited cooperation between clinical and spiritual teams; Perception that spiritual care conflicts with medical practice
	Poor external coordination	Insufficient collaboration with external institutions (e.g., municipality, media); Inadequate accommodation for religious diversity
Educational challenges	Lack of effective education	Insufficient scientific training and seminars; Caregivers lacking practical expertise
	Lack of value in the educational system	No dedicated curriculum or courses; Weak integration between educational policy and practice

Detailed descriptions of the codes are provided in the Appendix

### Lack of belief

Establishing spiritual care in hospitals requires attention to cultural beliefs. Belief is an essential component of comprehensive and effective spiritual care [14]. Two subcategories were identified: lack of knowledge and awareness, and challenges of contextualization.

#### Lack of knowledge and awareness

This refers to insufficient awareness, incomplete understanding, and inadequate recognition of the concept, importance, and benefits of spiritual care. Both patients and clinical staff may not fully grasp what spiritual care entails or its effects on health and well-being. Individuals with limited knowledge may struggle to understand abstract concepts such as meaning in life, hope, and faith, which are central to spiritual care[15].

“…Unfortunately, people and medical staff think that spiritual care means telling people to be religious or enjoining good and forbidding evil….”

“…Hospital medical staff do not understand the central concept and purpose of spiritual care, nor are they aware of the nature and significance of the activities undertaken by the spiritual care unit….”

#### Challenges of contextualization

These challenges involve the difficulty of adequately conveying the concept of spiritual care and its benefits to diverse individuals. If caregivers cannot clearly explain what spiritual care is and how it can help, acceptance is likely to decrease [12].

“…Some people only look at a person’s body and think that’s enough. It’s as if health is only about the body and the soul, mind, and spiritual care are not a priority….”

“…In healthcare settings, one key barrier is the lack of a cultural context in which spiritual care can take root and thrive. This cultural vacuum makes implementation difficult….”

“…They have a rigid, ideological view of spiritual care. They think there’s only one right way to do it….”

### Inefficiency in resource management

Resource management is critical to the success of spiritual care programs. Challenges were identified in three areas: infrastructure, financial allocation, and human resources.

#### Infrastructure challenges

Implementing programs in hospitals requires adequate facilities and equipment. Logistical shortcomings should not be borne solely by executive staff.

“…In public hospitals, the presence of communal spaces compromises patient privacy; there needs to be a dedicated area where patients feel safe to discuss their concerns….”

“…To obtain the tools and equipment we need, we must run around a lot; the process takes too long, and this unit is not given a budget….”

#### Lack of allocation of financial resources

Limited financial resources hinder planning and implementation. Interviewees highlighted the absence of specific budget allocations at the national level and insufficient prioritization of spiritual care at the provincial level.

“…There is a lack of proper budgeting. Occasionally, equipment and resources require lengthy approval and procurement. If the ministry allocated a specific quarterly budget to universities, we could plan more effectively….”

“…Irregular or non-payment of salaries to spiritual care providers is a serious challenge, reducing their available working hours….”

#### Human resource challenges

A sufficient number of qualified personnel is necessary for program success. Key issues included the absence of permanent spiritual care staff, a shortage of academically trained clergy and specialists, recruitment and retention problems, and an overall lack of spiritual care experts.

“…In most hospitals, there are clerics who do not have academic training in spiritual care, and we have limitations in this area….”

“…It is difficult to attract specialists, and even if they come, they do not stay. Clergy turnover is frequent…”.

### Process challenges

Process challenges concern actions designed to enhance, control, and monitor organizational procedures [17, 18]. Interviewees identified weaknesses in laws and regulations, lack of enforcement guarantees, management instability, and absence of a documented monitoring and evaluation system.

#### Weaknesses in laws and regulations

Effective implementation requires clear, communicated laws and procedures for referral, service provision, follow-up, and documentation. Without them, confusion, duplication, and lack of coordination may occur.

“…The lack of a dedicated organizational chart and hierarchy at the ministry level indicates that spiritual care does not have a formal place in the health system….”

“…The lack of a specific referral protocol causes confusion for patients and providers…”.

#### Lack of enforcement guarantee

Absence of enforcement mechanisms can undermine implementation. While managerial support can facilitate progress, inconsistent engagement from policymakers and managers impedes continuity.

“…When the manager is aligned with us, we can advance quickly; otherwise, progress stops entirely….”

“…Our meetings are too infrequent. The hospital director should regularly convene discussions with departmental physicians and experts in mental health and social sciences regarding spiritual care….”

#### Lack of management stability

Frequent changes in upper management disrupt program goals and hinder continuity.

“…The existing system does not formally recognize the spiritual dimension as vital. With changes in government, spiritual care initiatives often face closure or disruption….”

“…The management approach to spiritual care is unstable; sometimes it is pursued with enthusiasm, sometimes it is forgotten…”.

#### Lack of a documented monitoring and evaluation system

Monitoring and evaluation are essential for assessing the impact of spiritual care. Although outcomes may be difficult to measure directly, systematic mechanisms are needed to evaluate effectiveness and improve service quality.

“…The lack of continuous and systematic monitoring mechanisms challenges accurate assessment of service quality and prevents identification of sustainable strengths and weaknesses….”

“…Despite implementation efforts, we have seen insufficient documented and reliable results in previous projects, making it difficult to measure true effectiveness….”

### Limitations of program organization

Effective program organization requires cooperation and communication within and across departments and with external systems. Participants noted poor intra- and inter-sectoral coordination.

#### Poor intra-sectoral coordination

Acceptance and collaboration from clinical personnel are essential for spiritual care. Strengthening teamwork within the spiritual care team is foundational.

“…The most significant barrier is a lack of constructive dialogue and some negative perceptions; many believe spiritual care interferes with medical practice. On the contrary, spiritual care supports medical work….”

“…Lack of ongoing communication between physicians and the spiritual care team means the team is often unaware of patient condition details, making coordinated support difficult…”.

#### Poor external coordination

Collaboration with external organizations—such as municipalities, the Iranian Broadcasting Corporation, seminaries, and counseling centers—could raise awareness and support hospitals in establishing spiritual care systems.

“…Cooperation among institutions outside the university is essential. Meetings should be held with leadership to emphasize that this issue is not limited to hospitals….”

“…Neglecting attention to minority religions, such as Sunni and Zoroastrian communities, may hinder the effectiveness of spiritual care….”

### Educational challenges

Educational programs for clergy, caregivers, and executives can support professional development and improve spiritual care implementation.

#### Lack of value in the educational system

Participants emphasized that spiritual care must be recognized and valued as a core component of nursing and medical education. Without curricular recognition, successful implementation is unlikely.

“…For spiritual care to achieve its rightful place, a dedicated curriculum must be established outlining necessary knowledge and skills for students….”

“…Training for healthcare providers should focus on practical skills across physiological, pathological, psychological, and spiritual domains….”

#### Lack of effective education

Challenges in training include insufficient scientific instruction, inadequate seminars, providers lacking expertise, and a lack of awareness about relevant rules and protocols.

“…Seminars and conferences in this area are not held for medical staff and specialists. It is as if a charging station for the mind and soul is needed, but it does not exist….”

“…Specialized and ongoing training for all caregivers is essential to enhance knowledge, improve communication, and foster understanding of patients’ emotional and psychological needs….”

“…It is essential to provide specialized and ongoing training for all caregivers. This training should be designed to enhance specialist knowledge, improve communication skills, and foster mutual understanding of the emotional and psychological needs of patients, so that we can see an integrated and effective approach at all levels of service delivery….”

## Discussion

Spiritual care is an essential component of holistic healthcare, yet its implementation faces persistent multi-level barriers. Our study identifies four interrelated domains of obstacles—belief and awareness, resource management, process/governance, and organization/coordination—each of which constrains the recognition, provision, and sustainability of spiritual care in Iranian clinical settings.

Compared with international evidence, many barriers we observed are common worldwide. Ambiguity in the definition of spiritual care, cultural and belief differences, and inadequate education have been repeatedly documented in systematic and comparative studies [5, 21, 22]. In line with these reports, our participants emphasized unclear conceptual boundaries and low awareness among patients and staff as foundational problems that reduce acceptance and uptake of services [23, 24]. This supports the need for nationally coordinated awareness and definitional clarity as a prerequisite for broader implementation.

Contextual and cultural factors amplify these general problems in Iran. The country’s religious and ethnic diversity, together with specific Iranian–Islamic norms, shape how spiritual care is understood and accepted. For example, when spiritual care is presented mainly as religious ritual, many patients fail to recognize its broader role in providing meaning, hope, and comfort [19, 25, 26]. Successful interventions must use culturally sensitive framing and communication strategies. Staff training should help explain and adapt spiritual care to diverse belief systems [27, 28].

Resource and infrastructure limitations are central practical barriers. Participants reported shortages of private spaces, insufficient budgets, and a lack of full-time, trained personnel—constraints that mirror findings from international studies [16, 29–31]. Addressing these requires both dedicated funding and organizational changes (formal job descriptions, stable staffing, and physical facilities) to move spiritual care from an optional to an integrated service.

Weak governance and absent monitoring systems further hinder consistent delivery. Legal ambiguity, managerial instability, and the lack of an enforced accreditation or evaluation framework lead to fragmented and unreliable programs. Prior analyses stress the importance of documented evaluation and system-level planning for sustainable implementation [32–34]. Establishing clear legal responsibilities and an oversight mechanism would therefore help institutionalize spiritual care and reduce disruption caused by managerial turnover.

Educational shortcomings perpetuate low preparedness among healthcare providers. The omission of spiritual care from core curricula, the scarcity of standardized training, and insufficient practical instruction were repeatedly noted by participants and align with national and international reviews [18, 35–39]. Integrating competency-based training, practical workshops, and continuing education—developed in collaboration with experts—would strengthen providers’ ability and willingness to deliver spiritual care [7].

Finally, organizational coordination—both intra- and inter-sectoral—remains weak. Better integration across departments and stronger partnerships with external religious, social, and charitable organizations can extend capacity, raise public awareness, and provide referral support, as suggested by recent national proposals [33–35, 40]. In summary, our findings suggest that piecemeal solutions are inadequate. Effective implementation requires a multifaceted strategy that simultaneously addresses definitional clarity, education, resources, governance, and coordination.

## Conclusion

### Summary of findings

This qualitative study identified multi-level barriers to implementing spiritual care in Iran. Key findings are: (1) Limited public and professional understanding narrows the perceived scope of spiritual care; (2) Resource shortages (spaces, budgets, trained personnel) impede service delivery; (3) Weak legal and managerial frameworks and absent monitoring systems produce inconsistent implementation; (4) Educational gaps leave providers ill-prepared to address spiritual needs.

### Stakeholder perspectives

- Patients: Many view spiritual care narrowly (often as religious ritual), and fear of stigma limits disclosure of spiritual needs.
- Healthcare providers: Recognize the value of spiritual care but cite inadequate training, unclear roles, and resource constraints that make provision difficult.
- Managers/policymakers: Structural deficiencies—absence of formal roles, legislation, and monitoring—undermine sustainable program deployment.

### Limitations

The study’s qualitative design yields in-depth, context-specific insights most applicable to the Iranian setting; findings should be generalized with caution. Future comparative research across religious and cultural groups and quantitative evaluation of intervention effects would strengthen external validity.

### Policy recommendations (actionable)

Legal and governance reform: Define spiritual care, patient rights, and institutional responsibilities through national legislation and establish an independent oversight or accreditation mechanism to ensure continuity and enforcement.

Resource and infrastructure investment: Allocate dedicated budget lines, include standards for private and safe spaces in facility design, and formalize full-time positions with clear job descriptions for spiritual care personnel.

Educational reform: Integrate spiritual care competencies into undergraduate and postgraduate curricula, implement standardized practical training and in-service workshops, and establish specialized training centers at universities.

Quality assurance and information systems: Develop documented monitoring and evaluation frameworks and a national information system to collect indicators that support continuous improvement and research [28, 32, 35].

### Directions for future research

Evaluate the effectiveness of specific spiritual care interventions on patient-centered outcomes (quality of life, mental health), validate culturally adapted models of care, and develop standardized assessment tools for spiritual needs and care delivery. Comparative and mixed-methods studies will help identify the most scalable and culturally appropriate strategies.

Implementation of these measures—simultaneously tackling definitional clarity, education, resources, governance, and coordination—offers the most promising route to embedding spiritual care into routine, patient-centered clinical practice and improving overall wellbeing.

## Appendix (detailed descriptions of the codes in Table 2)

- *Misunderstanding of spiritual care*: Staff and patients equate spiritual care with proselytizing or simply telling patients to be more religious; limited grasp of its purpose and scope.
- *Staff unaware of spiritual care unit role*: Clinical teams unfamiliar with unit functions and referral pathways.
- *Failure to create cultural context*: Lack of localized messaging and cultural framing that would make spiritual care acceptable and relevant.
- *One-dimensional (biomedical) view of health*: Emphasis on physical health only; neglect of psychosocial and spiritual dimensions.
- *Ideological rigidity about spiritual care*: Belief that there is one “correct” way to deliver spiritual care, reducing adaptability.
- *Insufficient private/appropriate spaces*: No dedicated, private areas for spiritual conversations; communal wards compromise privacy.
- *Bureaucratic delays for equipment/supplies*: Long procurement processes impede service provision.
- *No dedicated budget for spiritual care*: Absence of line-item funding at national/provincial levels.
- *Irregular or insufficient payment to staff*: Delays or nonpayment reduce staff motivation and availability.
- *Shortage of qualified clergy/experts*: Lack of academically trained spiritual care professionals.
- *Recruitment and retention difficulties*: High turnover and difficulty attracting specialists.
- *No formal MOH organizational placement*: Spiritual care has no clear place in ministry organizational charts.
- *Undefined referral protocols*: Unclear who refers, when, and how patients access spiritual care.
- *No dedicated MOH unit*: Absence of a central coordinating unit at ministry level.
- *Insufficient managerial/administrative support*: Variable managerial engagement weakens implementation.
- *Irregular policy meetings and follow-up*: Lack of routine coordination and oversight meetings.
- *Program disruption after leadership change*: New managers may deprioritize existing initiatives.
- *Lack of sustainable organizational culture*: No institutionalization of spiritual care practices.
- *No systematic monitoring/evaluation*: Lack of indicators and processes to assess quality and outcomes.
- *Lack of documented evidence of program effectiveness*: Previous projects lack reliable documented results.
- *Limited cooperation between clinical and spiritual teams*: Communication gaps and poor sharing of patient information.
- *Perception that spiritual care conflicts with medical practice*: Misconceptions that spiritual care interferes with clinical care.
- *Insufficient collaboration with external institutions*: Poor partnerships with municipalities, media, seminaries, counseling centers.
- *Inadequate accommodation for religious diversity*: Limited attention to minority faiths (e.g., Sunni, Zoroastrian).
- *Insufficient scientific training and seminars*: Few continuing education opportunities focused on practical skills.
- *Caregivers lacking practical expertise*: Providers lack hands-on training for spiritual assessments and interventions.
- *No dedicated curriculum or courses*: Spiritual care not embedded in undergraduate/graduate curricula.
- *Weak integration between educational policy and practice*: Educational planning not aligned with clinical needs.

## Supplementary Information

Supplementary Material 1.

Supplementary Material 2.

## Data Availability

Data availability: The full text of the interviews is available and can be accessed by the corresponding author upon request.

## Abbreviations

HCPs: Healthcare Professionals
MAXQDA: Max Qualitative Data Analysis software
QoL: Quality of Life
PTSD: Post-Traumatic Stress Disorder
COVID-19: Coronavirus Disease 2019
ICU: Intensive Care Unit
IR.SSU.SPH.REC: Ethical Committee Code of Shahid Sadoughi University
NGO: Non-Governmental Organization
DSM: Diagnostic and Statistical Manual of Mental Disorders
PRO: Patient-Reported Outcome
