# Supplementary material for: Challenges of providing spiritual care to patients in Iranian teaching hospitals: a qualitative study
Source: BMC Psychol. 2026 Jan 26;14:354. doi: 10.1186/s40359-026-04028-0 (PMC12997921; doi:10.1186/s40359-026-04028-0)
Supplement: Supplementary file 1 — Supplementary Material 1. [file 40359_2026_4028_MOESM1_ESM.pdf]

Consent to participate in the Identifying the Challenges of Implementing Spiritual Care in Teaching Hospitals of Shahid Sadoughi University of Medical Sciences, Iran

Dear Sir/Madam

You are hereby invited to participate in the above-mentioned research. Information related to this research is provided in your service sheet and you are free to participate or not to participate in this research.

You are not obliged to make an immediate decision and to make a decision in this regard; you can ask your questions to the research team and consult with any expert you wish. Before signing this consent form, make sure that you have adequately and appropriately understood all the information in this form and that all your questions have been answered.

Project Administrator

-١I have been informed that the objectives of this research are: Identifying the challenges of implementing the spiritual care program in health centers and receiving suggestions for improvement

....

....

-٢I have been informed that my participation in this research is completely voluntary and I am not obliged to participate in this research. I was assured that if I did not agree to participate in the study, I would not be deprived of routine diagnostic and therapeutic care. My therapeutic relationship with the medical centers and staff would not be disrupted, and my usual treatment methods would continue.

-٣I was informed that even after agreeing to participate in the study, I could withdraw from the study at any time. My withdrawal would not deprive me of routine diagnostic and therapeutic care and would not require payment of a fine or compensation.

-۴My participation in this study is as follows: Participation in an interview...............
No specific complications

-٧I am aware that in case of severe possible complications resulting from my participation in the research, the drug and method under study will be discontinued and the researcher will be responsible for compensating for the damages and its cost.

-٨I am aware that the people involved in this research will keep all information about me confidential and are only allowed to publish the general and group results of this research without mentioning my name and details.

-٩I am aware that none of the costs of conducting research interventions as follows will be borne by me: none.

-۱۰I am aware that if there is a change in the research process that affects my health and decision to continue participating in the project, I will be informed.

-۱۱I am aware that in some circumstances, such as lack of interest in cooperation, my participation in the study may be terminated.

-۱۲I am informed that the implementers will inform me of the results of this project after the completion of the research.

-۱۳I am informed that if I have any problems or objections to the participants or the research process,

I can contact the Research Ethics Committee of the School of Public Health, Shahid Sadoughi University of Medical Sciences, Yazd, Iran at +98-03533123009 and raise my problem.

-۱۵Dr./Ms. Dr. Najmeh Baghian was introduced as the implementer of the project to answer me and I was told to discuss with him and ask for guidance whenever I have any problems, questions or unwanted complications regarding participation in this research.

Mobile phone: 9132742596 Landline: .... E-mail address: n.baghian@yahoo.com

Therefore, I, ..., child of .... with ID number .... and national code .... Considering the above and having obtained sufficient information, hereby declare my informed and voluntary consent to participate in the above research project.

Address and contact number :

....

Participant’s signature and fingerprint:

Date:
