# Supplementary material for: Challenges of providing spiritual care to patients in Iranian teaching hospitals: a qualitative study
Source: BMC Psychol. 2026 Jan 26;14:354. doi: 10.1186/s40359-026-04028-0 (PMC12997921; doi:10.1186/s40359-026-04028-0)
Supplement: Supplementary file 2 — Supplementary Material 2. [file 40359_2026_4028_MOESM2_ESM.pdf]

The interview was developed for this study.

The main and initial interview questions include:

1. Challenges in providing spiritual care in hospital

2. Infrastructure required for providing spiritual care in hospital

3. Facilitators of providing spiritual care in hospital

4. Obstacles in providing spiritual care in hospital

5. Strategies for promoting, implementing and enforcing the spiritual care system in hospital
